# Real-Time Patient Survey Data During Routine Clinical Activities for Rapid-Cycle Quality Improvement

**DOI:** 10.2196/medinform.3697

**Published:** 2015-03-12

**Authors:** James Lucius Wofford, Claudia L Campos, Robert E Jones, Sheila F Stevens

**Affiliations:** ^1^Wake Forest UniversityWinston-Salem, NCUnited States; ^2^Wake Forest Baptist HealthWinston-Salem, NCUnited States

**Keywords:** quality improvement, patient-centered care, information management/informatics, office practice issues/practice reengineering

## Abstract

**Background:**

Surveying patients is increasingly important for evaluating and improving health care delivery, but practical survey strategies during routine care activities have not been available.

**Objective:**

We examined the feasibility of conducting routine patient surveys in a primary care clinic using commercially available technology (Web-based survey creation, deployment on tablet computers, cloud-based management of survey data) to expedite and enhance several steps in data collection and management for rapid quality improvement cycles.

**Methods:**

We used a Web-based data management tool (survey creation, deployment on tablet computers, real-time data accumulation and display of survey results) to conduct four patient surveys during routine clinic sessions over a one-month period. Each survey consisted of three questions and focused on a specific patient care domain (dental care, waiting room experience, care access/continuity, Internet connectivity).

**Results:**

Of the 727 available patients during clinic survey days, 316 patients (43.4%) attempted the survey, and 293 (40.3%) completed the survey. For the four 3-question surveys, the average time per survey was overall 40.4 seconds, with a range of 5.4 to 20.3 seconds for individual questions. Yes/No questions took less time than multiple choice questions (average 9.6 seconds versus 14.0). Average response time showed no clear pattern by order of questions or by proctor strategy, but monotonically increased with number of words in the question (<20 words, 21-30 words, >30 words)—8.0, 11.8, 16.8, seconds, respectively.

**Conclusions:**

This technology-enabled data management system helped capture patient opinions, accelerate turnaround of survey data, with minimal impact on a busy primary care clinic. This new model of patient survey data management is feasible and sustainable in a busy office setting, supports and engages clinicians in the quality improvement process, and harmonizes with the vision of a learning health care system.

## Introduction

Soliciting and using patients’ ideas about how to improve health care delivery is an essential part of a patient-centered health care system [1-3]. Collecting patient’s ideas can be as simple as a waiting room suggestion box or a focus group of active patients. The patient survey is another easy tool for obtaining input from a larger population of health care users. However, patient surveys have increased in length and complexity to the point that many institutions have outsourced the survey conduct and data management to third party vendors or governments. Disadvantages of outsourcing the patient survey include recall bias for the patient, delay in data turnaround, and inadequate number of patient responses. Most important among these disadvantages perhaps is the disengagement of clinicians and staff from the quality improvement process [4].

The Plan-Do-Study-Act (PDSA) method is a rapid-cycle quality improvement process borrowed from other industries that suits the rapid pace of the clinical setting and the need for rapid turnaround of quality data [5-7]. The ground-up strategy of the PDSA cycle requires clinicians and staff to be more involved in quality improvement, but time is at a premium in a busy clinical setting, especially for clinicians. In order to garner support from busy clinicians, rapid cycle improvements need to be more efficient and take full advantage of established and emerging technologies for data management.

This report showcases four patient surveys that we conducted in a primary care clinic over a one-month period using commercially available technology (Web-based survey creation, deployment on tablet computers, cloud-based management of survey data, and real-time Web-based accumulation and display of survey results) to expedite and enhance several steps in data collection and management for rapid improvement cycles. The four patient surveys targeted diverse yet specific areas for quality improvement and used a variety of personnel and deployment strategies. This approach of technology-enabled, rapid cycle improvement offers promise to clinics faced with limited budgets and time, and finally suggests a strategy of patient survey data management that is sustainable and accommodates the rapid pace of contemporary health care delivery.

## Methods

### Setting

Located in Winston-Salem, North Carolina, USA, the Downtown Health Plaza Adult Medicine Clinic is staffed by physicians and mid-level practitioners from Wake Forest University Baptist Health. The clinic typically logs more than 60,000 clinic visits each year and serves a large number of Medicaid and uninsured patients. Approximately 10% of patients are Spanish-speaking only. Four physician assistants have half-day clinic sessions every day, and 8 faculty and 40 resident physicians have clinic sessions 1-3 times a week. The EPIC electronic medical record serves as the data repository for all clinical information. Approval for this project was not initially sought from the local ethics review board as this is part of the clinic’s standard and mandated quality improvement process, and no patient identifiers were collected. Still, institutional review board approval was sought, and this project has been declared exempt.

Clinic staff have long been interested in quality improvement and population-based approaches to improving clinical care. Earlier efforts at rapid data collection of patient survey data during routine clinical activities made use of pre-printed index cards with 3-4 multiple choice questions that were completed by staff and providers [8]. The availability of multiple handheld and tablet computers, and the expertise at developing applications for those devices set the stage for piloting a new patient survey strategy using the tablet computers.

### Technology

CareToTell (Cupertino, CA, USA) is a commercially available Web-based suite of integrated data management tools that allows easy Web-based creation of survey forms, deployment and data collection using tablet computers, and real-time data accumulation and display on the company-hosted webpage. Sequential offerings of the survey on a single tablet computer is possible after an 8 second automatic reset, and survey deployment/conduct can occur on multiple tablets simultaneously. The data display on the website offers statistics on the average amount of time for the user to complete the entire survey and for each individual question; specific times for each respondent are only available for the entire survey, not for individual questions. Whether the survey was aborted, and which questions were skipped by the respondent is also available at the report webpage.

### Survey Deployment

Over the one month period, we conducted a total of four surveys. Nurses and secretaries serving as survey proctors were trained simply by taking the survey once, and then discussing the positioning of the tablets in the clinic and protocol for offering the survey to the patient. The nurses proctored 2 of the 4 surveys, and two surveys were proctored by checkout personnel as the patient obtained their follow-up appointment before exiting the clinic. Anecdotally, patients needed help from the proctor in a minority of cases; however, patients who were clearly unable to complete the survey because of language, literacy, or disability were often not offered the survey. This judgment was made by the proctoring staff member. Family members were allowed to complete the survey for patients who were unable, and the nurse occasionally helped patients with the survey.

### Survey Design

Each survey consisted of a maximum of 3 questions to minimize the burden that data collection would impose on the patient and on clinic workflow. Because of the limited number of questions and our decision to avoid patient identifiers, we chose not to solicit information on demographics or other patient characteristics that might affect patient confidentiality. We used only yes/no and multiple choice questions, and allowed survey questions to be skipped. For questions about continuity of health care, we drew from the CAHPS survey tool that is used in many health care settings to judge patient satisfaction [9]. We estimated that 1 to 2 days of data collection would be sufficient to accumulate enough responses to be credible and actionable.

### Data Analysis

We estimated the denominator for possible respondents by comparing the number of responses with the number of patients who attended clinic appointments on survey days. We characterized surveys by the percentage of the surveys that were aborted or contained skipped questions, and by the amount of time spent with each survey (mean, standard deviation, range). Average times were calculated after elimination of unrealistically short survey times. We characterized the average amount of time spent on each question by question type, order of question, proctor strategy, and number of words in the question. As this was a feasibility study, we did not attempt to establish an *a priori* sample size calculations and specific hypotheses to be tested.

## Results

### Setting

Of the 727 patients who attended office visits when one of the four surveys was deployed, 316 patients (43.4%) attempted the survey, and 293/727 (40.3%) ultimately successfully completed one of the four surveys.

### Data Analysis

Table 1 shows question formats, proctor strategy, completion rates, and the average times per survey.

For the 4 three-question surveys the average time per survey was overall 40.4 seconds, with the range of average answer times for individual questions of 5.4 to 20.3 seconds (Table 2). Yes/No questions took less time then multiple choice questions (average 9.6 seconds versus 14.0). Average response time showed no clear pattern by order of questions or by proctor strategy, but a trend was apparent for number of words in the question (>30 words - 16.8 seconds, 21-30 words - 11.8, <20 words - 8.0).

**Figure 1:**
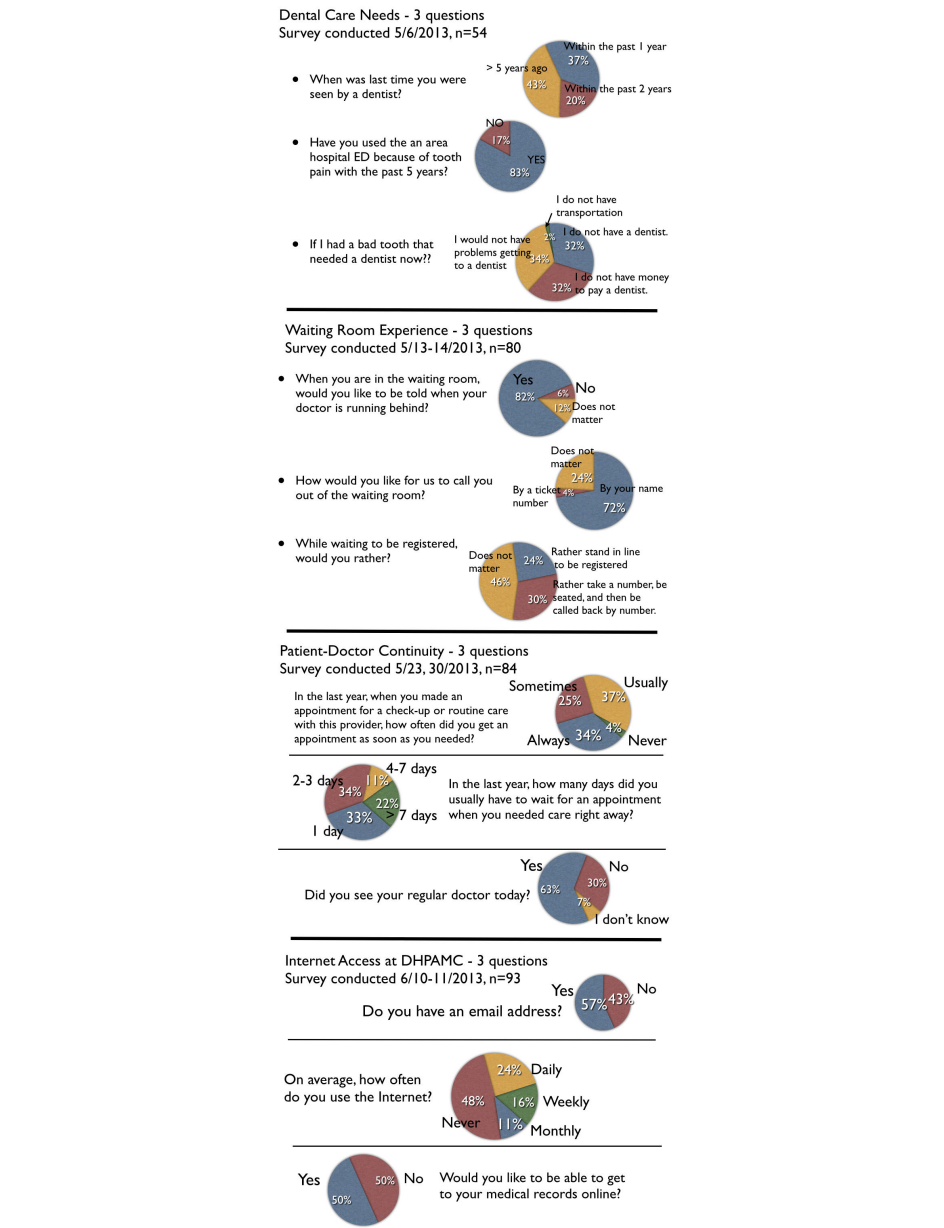
Survey findings from four patient surveys.

Figure 1 shows the findings of the four surveys that were presented to the clinic staff meeting for discussion, disseminated to the rest of the clinic, and policy making.

**Table 1:** Summary of the four patient surveys.^a^

Survey domain	Question formats	Proctor strategy	Patients completing survey, n (%)	Completions/ patients with clinic visit	Survey time, mean (SD)
Dental care	1 Yes/No 2 multiple choice (4 responses)	Secretary at clinic checkout and exit	54/56 (96.4)	54/110 (49.1)	42.2 (19.4)
Waiting room experience	1 Yes/No 1 multiple choice (3 responses) 1 multiple choice (4 responses)	Nurse in exam room	74/81 (91.3)	74/193 (38.3)	43.9 (31.0)
Continuity	1 Yes/No 1 multiple choice (4 responses) 1 multiple choice (5 responses)	Secretary at clinic checkout and exit	79/86 (91.8)	79/23 (33.9)	38.7 (24.8)
Internet access	2 Yes/No 1 multiple choice (4 responses)	Nurse in exam room	86/93 (92.3)	86/201 (42.8)	37.9 (21.8)

^a^ surveys with no skipped answers or aborted attempts

**Table 2:** Average respondent time by individual question.

Survey domain	Question	Question types^a^	Word number (stem + responses)	Average question time
Dental care	1. When was last time you were seen by a dentist?	MC4	10+15= 25	12.2
	2. Have you used the Emergency Room at area hospitals because of tooth pain with the past five years?	Y/N	20+2= 22	8.6
	3. If I had a bad tooth that needed a dentist now	MC4	11+29=40	19.5
Waiting room	1. When you are in the waiting room, would you like to be told when your doctor is running behind?	Y/N	19+5=24	16.2
	2. How would you like for us to call you out of the waiting room?	MC4	14+10=24	10.2
	3. While waiting to be registered?	MC3	5+26=31	14.8
Continuity	1. In the last year, when you made an appointment for a check-up or routine care with this provider, how often did you get an appointment as soon as you needed?	MC4	30+4=34	20.3
	2. In the last year, how many days did you usually have to wait for an appointment when you needed care right away?	MC5	22+14=36	12.5
	3. Did you see your regular doctor today?	Y/N	7+5=12	5.4
Internet access	1. Do you have an email address?	Y/N	6+2=8	9.5
	2. On average, how often do you use the Internet?	MC4	9+4=13	8.7
	3. Would you like to be able to get to your medical records online?	Y/N	13+2=15	8.5

^a^ MC=multiple choice; Y/N=yes/no

## Discussion

### Implementation Strategy and Impact

Our four patient surveys, conducted weekly over a one-month period, with different question types and deployment strategies, provided responses to important quality questions quickly enough to make the findings relevant and actionable. In the case of the dental care project, our findings were instrumental in justifying establishment of a community wide dental clinic for patients with poor dental access. With regard to the survey on waiting room experience, the findings led to revision of our current waiting room policies. The third survey on continuity between primary care physician and patient provoked important discussions about policies related to patient access and continuity, and also established a baseline for subsequent serial measurements that are increasingly required by accrediting organizations [10-12]. The last of the four surveys on Internet access repeated questions from our previous paper-based survey of Internet access [13], and addressed the increasingly important issue of patient willingness and ability to access online medical records. With all four surveys, the time from identification of the quality challenge and conception of survey questions to the time that results were disseminated to clinicians and administrators was less than one week, a time frame that encouraged rapid cycles of change in policy and health service delivery.

Underpinning our success with the four surveys was the overall response time per three-question survey of approximately 40 seconds. This rapid survey completion and turnaround time of the survey devices allowed the clinic to approach more patients in a busy clinic and yield a high response rate of 43%, a rate that rivals more sophisticated survey strategies [14-16]. The growing familiarity of patients/consumers with tablet computers along with supporting software should make this a preferred strategy for data demands in time pressed clinical settings [17]

### Limitations

Several caveats with this innovative approach to patient survey data and quality management deserve mention. The first concern of generalizability has several dimensions. First, not every patient could complete the survey. Low literacy is well known in our clinic, an issue we have documented many times [13]. Surveys were not attempted in Spanish, despite the ability of the software to provide translations. Furthermore, staff who were proctoring the survey process used judgment in offering the surveys to Spanish-speaking patients or patients with communication or cognitive disorders. Still, we were able to accumulate a sizable number of responses without fatiguing clinical staff and patients with ongoing surveys that can be disruptive to clinic flow. Our response rate of approximately 43% is a dramatic difference from the meager monthly offering of a handful of respondents who are often misattributed to our clinic by the third party patient surveyor. Even with our efforts to estimate a denominator for generalizability within our clinic, we instead depended on the large number of actually collected surveys to counter concerns of statistical significance and sample size. A comparison of survey respondents with nonrespondents might have been useful but would have hampered the survey administration and made the survey process less practical in a busy clinic. Generalizability beyond our clinic setting is a reasonable question. This clinic has long used tablet computers routinely in patient care activities for years, our wireless system is near flawless, and our faculty and staff have active interest in computer-assisted patient education and quality improvement.

A second caution relates to technical aspects of the software. The software performed well with regard to survey creation and deployment on the tablet computers, cloud capture of responses, and accumulation/display of data. Despite a usability and execution that was surprisingly good for a relatively new software product, the current version of the software does not allow the use of the graphics or video, which could enhance the accuracy and acceptability to low literacy patients. For analytics, the current version does not report user-specific, question-specific user times, a feature that impeded our ability to examine individual user question-specific interactions. However, this may be less important to clinics that simply want a tool to facilitate data gathering and reporting.

### Easier Data Collection and Management

The novelty presented here is not in the commitment of a community health care center aggressively pursuing quality improvement with rapid cycle projects. This rapid pace of practice improvement is evident in many health care settings where much time, energy, and manpower is being devoted to quality improvement, to the point of displacing regular clinical duties and wearying clinicians with change fatigue [18,19]. Rather, the novelty is the ease of the survey launch, the rapid data turnaround, and the time savings that more simple and practice-friendly data management brings to clinicians and administrators on the front line.

Making clinic operations patient-centered is a routinely stated goal of a modern health care delivery system and is important in settings as diverse as primary care and surgical theaters [12,20,21]. Data collection is a fundamental step in soliciting the patient’s opinion about how to improve health care delivery. If obtrusive, expensive, unrealistic, or inaccurate, the process of data collection can undermine enthusiasm for assessing and improving practice quality. Patients are already fatigued of requests for satisfaction surveys in health care and in daily life. Data collection must be quick, focused and part of routine care in order to encourage participation from patients and clinicians, and to be sustainable in a busy practice. Likewise, data entry and analysis are steps that can slow turnaround of quality improvement projects [17,22]. The commercially available technology suite (tablet computer, wireless environment, up-to-the-second, Web-based analytics) we used to conduct these four patient surveys is a major improvement over scannable paper survey forms, dedicated desktop computers for capturing patient survey data, and bulky, expensive waiting room kiosks from just a decade ago [16,23-25].

### A Learning Clinic

In order for clinicians to participate fully in quality improvement activities, practical, real-time strategies for reliable and more efficient data capture and management are needed [1]. Having good technology is only part of the answer. A strategy that focuses on specific patient care issues and timeliness of data collection enhances the accuracy of data capture [16]. Standardized, and well-tested questions are available for examining specific patient care domains [9]. Leadership and teamwork are still necessary in championing a new strategy that promotes the value of the quality improvement and data collection process, and creates an environment where rapid-cycle quality improvement becomes routine.

Improvements in technology and redesign of patient survey strategy make the goal of the learning health care system (or clinic) realizable [7]. Whether or not patient opinions have a legitimate role in quality assessment of a practice or health system is still being debated [26,27]. However, having a system of collecting patient opinions and ideas about improving health care delivery shows patients the interest and abilities of the practice clinicians in improving health care delivery, and facilitates engagement of clinicians in designing the quality improvement process. Regular patient surveys, now becoming routine in our practice, point to a sustainability that was simply not possible before the availability of this technology. Especially with mandates from accreditation agencies that clinical practices routinely monitor patient satisfaction in specific care domains, repeat questions from sequential surveys to track improvement is finally realistic [28].

### Conclusions

In a busy urban primary care clinic, the use of tablet computers and integrated data management software accelerated the usually burdensome task of surveying patients for quality improvement, and accomplished it during routine clinical activities. This practical implementation of technology-enabled, rapid-cycle quality improvement demonstrated a rapid turnaround time in the surveying process and in project completion, and showed a high response rate compared with other survey methodologies. Rapid-cycle quality improvement requires such a nimble data collection and management strategy in order to make the busy clinical setting a “learning” health care system.
